# Implantable Microimagers

**DOI:** 10.3390/s8053183

**Published:** 2008-05-15

**Authors:** David C. Ng, Takashi Tokuda, Sadao Shiosaka, Yasuo Tano, Jun Ohta

**Affiliations:** 1 Graduate School of Materials Science, Nara Institute of Science and Technology, 8916-5 Takayama, Ikoma, Nara 630-0192, Japan; 2 Graduate School of Biological Sciences, Nara Institute of Science and Technology, 8916-5 Takayama, Ikoma, Nara 630-0192, Japan; 3 Department of Ophthalmology, Osaka University Graduate School of Medicine, 2-2 Yamadagaoka, Suita, Osaka 565-0871, Japan; 4 CREST, Japan Science and Technology Agency, 3-5 Sanban, Chiyoda, Tokyo 102-0075, Japan

**Keywords:** implant, head, microimager, retinal prosthesis, *in vivo*

## Abstract

Implantable devices such as cardiac pacemakers, drug-delivery systems, and defibrillators have had a tremendous impact on the quality of live for many disabled people. To date, many devices have been developed for implantation into various parts of the human body. In this paper, we focus on devices implanted in the head. In particular, we describe the technologies necessary to create implantable microimagers. Design, fabrication, and implementation issues are discussed vis-à-vis two examples of implantable microimagers; the retinal prosthesis and *in vivo* neuro-microimager. Testing of these devices in animals verify the use of the microimagers in the implanted state. We believe that further advancement of these devices will lead to the development of a new method for medical and scientific applications.

## 1. Introduction

Half a century ago, the idea of having a device implanted in the body which is capable of sending out electrical pulses to stimulate the heart muscles resides in the realm of science fiction. Patients with medical conditions such as irregular heart beats were confined to the hospital bed and constantly monitored for survival. To save these patients, a revolutionary device was developed. About 50 years ago, the implantable cardiac pacemaker has helped patients survive life threatening cardiac arrests [1]. Since then rapid advancement in implantable device technology has created many similar life-saving devices. These devices are often the last resort for patients with life-threatening situations.

**Figure 1.**
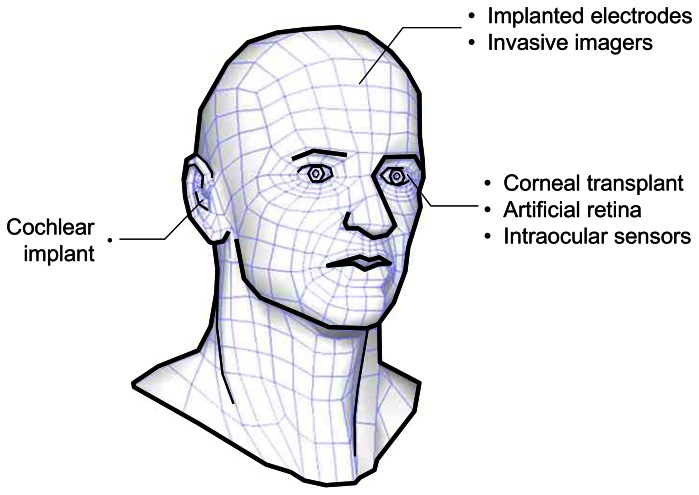
Implantable microdevices in the head.

Similarly, deaf and blind patients have to endure hardships due to their disabilities. Again, technology is able to give these patients relief in the form of artificial implant devices. One of the goals of these devices is to replace biological organs which have been degenerated by diseases or other causes. Figure 1 shows the various possibilities for bionic implants in the head. Sensing organs such as the ear and eye can, in theory, be replaced by an artificial device. Brain interface devices, on the other hand, are being explored to study functions of the brain.

By far, the most successful neuroprosthetic device is the cochlear implant [2-3]. This implant system is made up of two separate parts; the receiver-stimulator and electrodes, and the microphone, speech processor and transmitting coil. The receiver-stimulator converts the codes received by radio waves into electrical signals that are sent along the electrode array. The electrode array is implanted surgically into the cochlea of the patient. It receives electronic signals and stimulates the auditory nerve so that the brain perceives sound. The electrode array has tiny electrodes connected to the receiver-stimulator. The electrodes deliver different signals that represent loudness and pitch of sound by stimulating the appropriate nerve fibers that send signal to the brain.

Apart from the ear, implantable electrodes have been developed for several other niche applications like brain electrophysiological experiments and treatment of neurological disorders [4-6]. Coupled to the nervous system, these devices can be used to record electrical activities or for electrical stimulation. The intent of these devices are to help tetraplegia patients regain lost motor functions, or as a medical tratement of epilepsy and Parkinson's diseases. Initially, these devices consist of only a single stimulating electrode. Further advances have led to more and more electrodes being developed on a single device. On the signal processing front, advancement in implantable electrodes includes sophisticated on-chip amplifiers and signal processing capabilities [7-9].

Against the background of these implantable devices, *microimagers* are recently being explored for various medical and scientific applications [10-13]. A microimager is defined as image sensor array chip which is packaged into a ready to use module. An implantable microimager, for example, can be developed into an artificial retinal prosthetic device. Such a device has the potential to restore partial vision to patients with forms of blindness caused by a loss of photoreceptor cells. Another useful application of an implantable microimager is imaging the brain of a freely moving animal. Current brain imaging techniques such as magnetic resonance imaging (MRI) or positron emission tomography (PET) are unable to image moving bodies. In this paper, we focus on implantable microimager devices. In particular, we describe the enabling technologies for creating implantable microimagers in the head. We focus on two particular types of devices, one of which is the retinal prosthesis and the other being the on-chip brain imaging device. Beginning with a brief overview of these devices, we then discuss the technologies underlying these devices. The capabilities of these devices are verified by implanting these devices and performing preliminary tests in animals.

## 2. Microimagers

As described above, a microimager is essentially an image sensor chip which is packaged into a small minimally-invasive module. This module is ready to be integrated into a larger system for imaging and measurement. In other words, we strip the conventional microscope down to its most essential element which is the image sensor and work from bottom up. Because the technology used to produce image sensors also allow sensing in other domains such as capacitance, electrical potential, temperature to name a few, new applications can be explored by developing these so-called multi-purpose lab-on-chip measurement devices [14-22]. Lal [23] and Grayson [24] gave comprehensive reviews of the available technologies to fabricate these devices. Here, we concentrate on two applications for microimagers; the retinal prosthesis and on-chip *in vivo* imaging.

**Figure 2.**
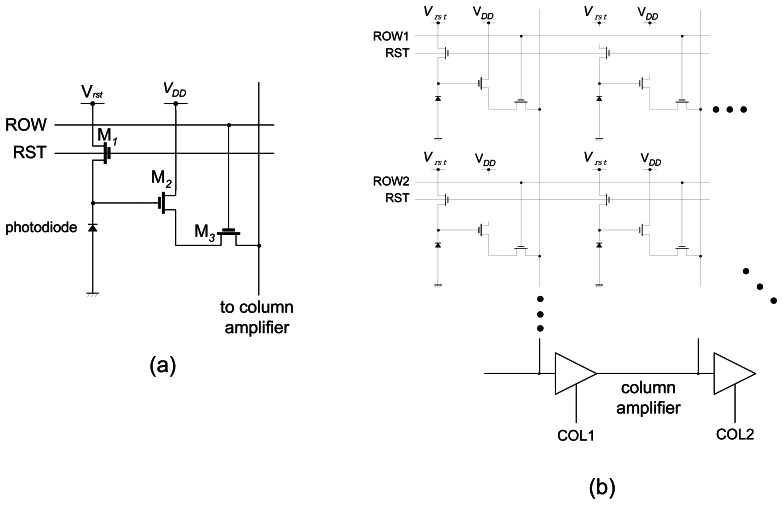
(a) Interconnection of photodiode and three transistor (M*_1_*, M*_2_*, M*_3_*) forming an active pixel sensor circuit. (b) Image sensor schematic based on three transistor APS circuit.

**Figure 3.**
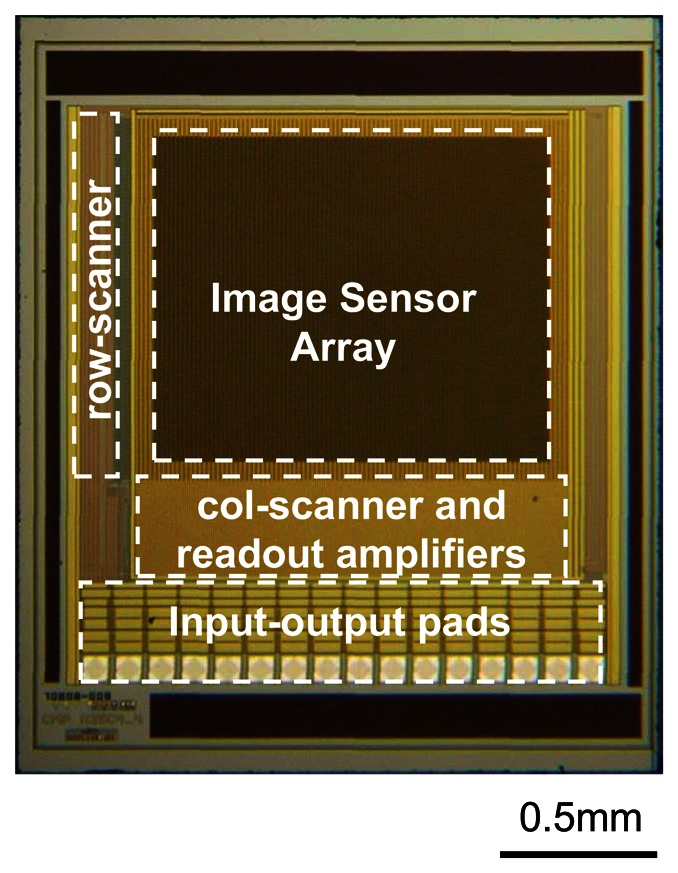
Typical microimager chip consisting of image sensor array, row and column scanners, and readout amplifiers (adapted from [40]).

The basic element inside a microimager is a photosensor. Using currently available CMOS-based semiconductor fabrication technologies, the size of a photosensor element can be as small as 5 μm. As the push for deeper submicron technologies advances, the pixel size will further reduce. Capitalizing on this advantage, the imaging resolution is expected to improve in the future. The materials used to fabricate a photosensor is doped Si. A physical combination of n-doped and p-doped Si forms a photodiode which acts as a convenient photosensor. Further, an array of photodiodes connected in a 2D fashion will form an image sensor array suitable for imaging. A common implementation of photodiodes interconnections for imaging is a three-transistor circuit often referred to as an Active Pixel Sensor (APS) shown in Figure 2 (a). A reset signal, RST is used to initiate the photodiode voltage and when light impinges on the photodiode, the photon energy is converted to electrical energy, and the photogenerated current will discharge the photodiode voltage. Using this scheme, a 2D array of interconnected APSs can be selected individually and their output signals can be read-out (Figure 2(b)). Careful timing of the ROW and COL clock pulses are necessary for correct operation of the image sensor. These clock pulses are usually generated using scanners or decoders. A typical image sensor chip and its specifications are shown in Figure 3 and Table 1 respectively.

Once fabricated, the microimager need to be carefully packaged for implantation inside tissue organs. Apart from interconnection, light source, filters, and final shape and size are important issues faced. Because these devices are implanted into living tissue over an extended period of time, sometimes over a year or more, many issues arise with their use. These include tissue inflammation and infection problems for the tissue; and reliability, power and data transmission for the device. These issues are encountered for both the retinal prosthesis and implantable on-chip imagers.

### 2.1. Retinal Prosthesis

Retinal prosthesis or artificial retina has been proposed to restore sight by direct electrical stimulation of the retina. Although other possible treatments are being investigated, the artificial retina implant chip has been reported as a potential solution for patients that are blinded by diseases such as retinitis pigmentosa and age-related macular degeneration [25]. In both cases, the natural photoreceptor cells of the eye are wasted. However, the rest of the vision system, including the optical nerve fibers up to the visual cortex remains intact. Hence, realizing the means to replace the degenerated photoreceptor cells artificially, gives hope of regaining visual sensation for patients inflicted by these irrecoverable eye diseases. Maynard [26] and Margalit *et al.* [27] surveyed the field of retinal prosthesis research in the early development phase. More recently, Weiland, Liu and Humayun reported on later progress of retinal prosthesis research [28].

In animal experiments, it was shown that phosphene or brief light sensation can be induced by electrical stimulation of the retina [29-31]. This paved the way for development of highly sophisticated 2D array of electrical stimulators, known as artificial prostheses. The prosthesis device can be implemented in several ways. A common implementation is a 2D array of electrodes which sends out electrical signals to the biological photoreceptors. The electrical pulse can be generated on-chip via an array of photodiodes or off-chip by using an external camera.

The retinal prosthesis consists of two main parts; an image sensor and a stimulus electrode array. One implementation decouples the image sensor and stimulus electrode [31-34]. Image data and power are transmitted from an external unit to the retinal implant. Another method is to combine these two parts into a single chip device [35-37]. The advantage of the latter is reduction in interconnections. Most of these devices can be fabricated using existing technology borrowed from the semiconductor industry. One important issue for implementing a microimager as a retinal prosthesis is flexibility. Because of the curvature of the eyeball, this adds a physical demand on the overall shape of the device. Currently ways to overcome this problem are being investigated [38].

### 2.1. On-chip In-vivo Microimagers

We first proposed and demonstrated *in vivo* imaging inside the mouse brain using a microimager in our previous work [39]. The immediate application for invasive imagers is to study the dynamics of chemical reactions inside the brain. Although the level of detail offered by these microimagers is below that of normal optical microscopes, they can be useful when sections deep inside the brain need to be imaged. Used in conjunction with other brain imaging systems such as magnetic resonance imaging and positron emission tomography, microimagers can reveal functions of the brain not easily accessibly by conventional microscopes. Moreover, by using an implanted device, the behaviour of a freely-moving animal can be observed without restraining it. These advantages are the main motivation factors for developing an on-chip *in vivo* imager. Apart from that, the on-chip imager has inherent high resolution spatial and temporal imaging capabilities [40-43]. Furthermore, with multimodal sensing capabilities, on-chip imaging offers an attractive tool for exploring body organs internally.

For this method the object of interest is directly coupled to the imager without intermediary optical elements. The advantage offered by on-chip imaging method is higher efficiency for collection of light. The resolution offered by this method is limited by pixel size as well as distance between object and sensor surface.

As a device used for implantation, the relative size of the microimager is an important criterion. Too big, and we run the risk of injuring the organ to be imaged. Too small and the original imaging intent is lost. Hence, we seek a right balance between device size and invasiveness. Apart from that, a light source is necessary for optical imaging. Effective methods to integrate a light source onto the microimager module are necessary to successfully develop an implantable optical microimager. Furthermore, in order to perform fluorescence imaging, a suitable optical filter is also required.

## 3. Fabrication of Microimagers

To develop practical implantable devices, precise and reproducible fabrication techniques and interfacing technologies are required. Fabrication of microimagers depends on the same technology which is used to produce highly integrated circuit (IC) chips. IC fabrication technologies are used to produce devices with dimensions in the micrometer or millimeter range and are capable of outputting thousands of devices using batch processing. Although there are many IC-related technologies, such as CMOS, bipolar and other processes involving specialized materials such as SiGe, current mass produced IC chips are based mainly on Si and are almost synonymous with CMOS technology.

A related technology which is used to fabricate small mechanical structures called microsystem technology (MST) or microelectromechanical systems (MEMS) borrows a variety of materials and processes from the IC field. In MEMS processes, etching and deposition of materials are used to selectively remove or add materials. Variations of these processes often referred to as bulk or surface micromaching, have been developed over the years to realize small sensors and actuators.

So much are in common between CMOS and MEMS processes that CMOS-based MEMS processes are becoming popular as a cost effective development tool for integrated microdevices. Combining CMOS and MEMS technologies offer many attractive advantages in the fabrication and realization of implantable microimagers. Overall, three fabrication methods are available to fabricate microsystems, and these processes are categorized by the entry stage of MEMS micromachinging into the IC process line. These micromaching steps can precede the standard CMOS process sequence (pre-CMOS), performed in-between CMOS process steps (intra-CMOS), or be performed after completion of the CMOS process (post-CMOS). An excellent review by Brand [44] provides a thorough overview of these technologies.

In our work, we capitalize on the services of multi-chip foundries, which enable us to develop chips at a fraction of the cost of running a full CMOS production facility. This is similar to traditional vendor services for mechanical parts, but at a higher technological level. Starting with the design of the CMOS image sensor chip, we send out the design to the multi-chip foundry. Once the device is fabricated and returned, we performed post-processesing to define the final device structure. Integration with LEDs and electrode formation completes packaging of the microimager module.

### 3.1 CMOS Microelectronics

Since the development of the first integrated circuits (ICs), it was quickly realized that IC can be integrated into micro devices thus forming implantable systems [45]. On-chip circuitry consists of a large number of transistor gates and interconnections, resistance, capacitors, and inductors.

**Figure 4.**
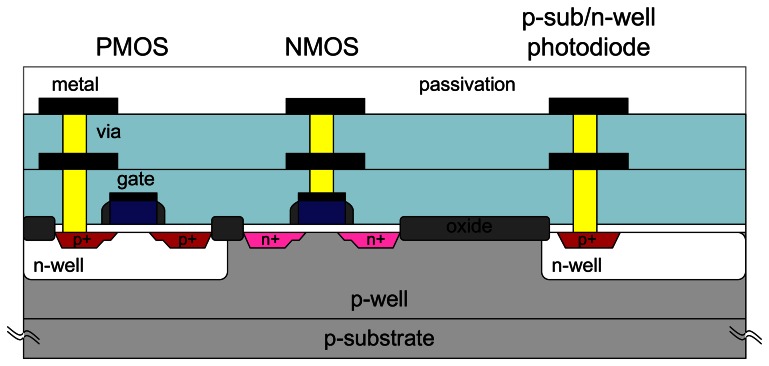
Simplified cross-section schematic of typical CMOS technology (0.5 - 1 μm) with two-level metal interconnections.

A detailed discussion on CMOS process is beyond the scope of this paper. However, for completeness, we review a typical device fabricated using conventional CMOS process. Figure 4 shows the cross-sectional view of a CMOS device. It consists of NMOS and PMOS transistors, and a photodiode. These devices are connected with multiple metal levels. The need for multilevel metal layers is apparent when device size shrinks and device interconnection increases. For a 2D pixel array, the minimum metal interconnection layers needed is two.

The microimager module can be completely fabricated using standard CMOS process. As mentioned, the main element of the microimager is a photosensor circuit called an active pixel sensor. It consists of three NMOS transistors and a photodiode. A 2D array of APSs is connected with multiple layers of metal interconnections forming the image sensor array. To conserve power consumption, the column amplifier is designed with both NMOS and PMOS transistors.

### 3.2 MEMS Fabrication

**Figure 5.**
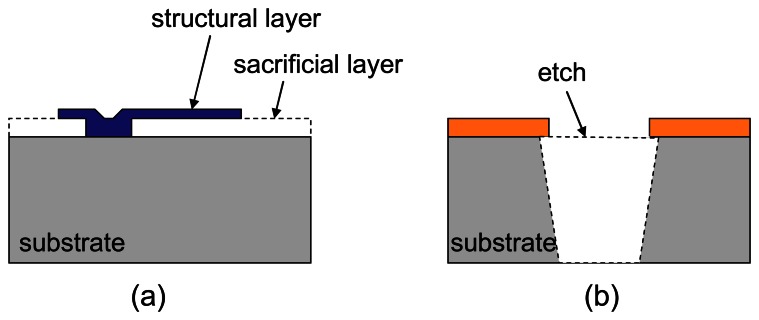
Schematic cross-section of device undergone (a) surface, and (b) bulk micromachining.

Traditionally, basic MEMS micromachining techniques include surface and bulk processes (Figure. 5). Combined, these processes are used to fabricate a host of mechanical sensors and actuators which include pressure and inertial sensors and interdigital comb drives [47]. Recently, the field of MEMS devices has grown to encompass many other sensors such as chemical, optical, thermal. However, not all MEMS processes are compatible with the standard CMOS process. Depending on the process flow, MEMS machining can occur before, during or after completion of the CMOS process.

We used a particular MEMS fabrication technique to post process the microimager chip by etching through the substrate. This MEMS bulk etching postprocess called Deep Reactive Ion Etching (DRIE) can be used to bore a fairly vertical hole, through the substrate if required. Using this method, we can define the shape of the device as well as open holes through the chip. This technique allows us to minimize injury by defining a hyperbolic curvature on the insertion surface of the module as well as permit illumination light to pass through the chip.

**Figure 6.**
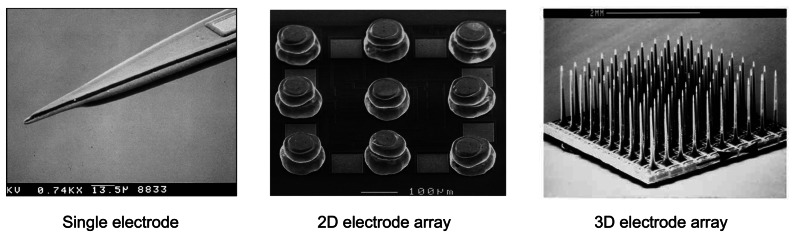
Examples of single electrode [47] (© 2004 IEEE), 2D electrode array [38], and 3D electrode array [48] (© 1999 IEEE) fabricated using MEMS micromachining processes.

MEMS fabrication processes are also utilized to form electrodes on a Si substrate. Due to the maturity of Si microfabrication process, it can be shaped with great precision and repeatability. Single electrodes, 2D and even 3D electrodes have been developed using Si as the base material [47-48]. Figure 6 shows examples of these microfabricated electrode devices. These devices are used mainly as neural prosthesis in electrophysiological recordings. Compatibility with standard CMOS process is an important requirement when considering the choice of available electrode fabrication technique. This is because a single chip solution will necessarily reduce the final size of device which is required for implantation.

### 3.3 Packaging Method

Packaging of implantable devices presents a number of special challenges. Even for physical microsensors, the challenges are non-tivial. Baltes [49] gave us some insights on the issues involved in packaging of microsensors. On top of protection for the implantable device, we are faced with additional issues such as biocompatibility, hermetic sealing, and interconnections. Furthermore, formation of electrodes and integration of illumination and chemical delivery system adds to these difficulties. For a retinal prosthesis device, forming an effective electrode on the device surface for electrical stimulation is essential. This is also true for an *in vivo* imager which is used for simultaneous stimulation and recording of neuron electrical activities. Currently, there are no general or standard packaging techniques available for implantable devices. Most packaging methods customized for their specific applications and are mostly developed based on trial and error.

Electrical field potential recording and current stimulation are the main ways to directly interface with neurons [50-51]. Apart from the types of materials used, the electrode shape and form of stimulus pulse is also important for successful stimulation of cells. Electrodes which can be fabricated with small area yet have large charge delivery capacity are most effective for cellular stimulation. Pt and Ir have been recognized to be excellent material for forming electrodes [52-54]. Apart from material selection, formation of electrode on the microimager device is also non-trivial. Several methods have been explored to form electrodes [55-57]. Chemical deposition and electroplating methods gives a durable electrode surface finish, however the electrodes form this way are recessed below the passivation layer of the device surface. This leads to inefficient contact with cells. On the other hand, electrodes fabricated by physical metal evaporation are not stable and do not last though the duration of chronic experiments. We found that forming an electrode by supersonic vibration at elevated temperature gives a very durable electrode [58]. Using this method, the choice of materials is not limited by process availability. By using a modified wire bonder, electrode bumps can be formed. Also the size of the electrode including height and diameter can be controlled.

Fluorescence imaging is a method which allows visualization of changes at the molecular level using chemical probes. These probe molecules or markers, can be used to detect the presence of specific molecules once bound to its specific target to form a fluorescence compound. Fluorescence occurs when the resultant molecule is excited by an incident light at a lower wavelength, and generates light energy at a higher wavelength when it relaxes back to its original state. This phenomenon is called the Stokes shift. In order to perform fluorescence imaging, three elements are necessary; an imager, a light source, and a light filter. Integration of all these elements onto a single chip is necessary to perform *in vivo* imaging. Light-emitting diodes (LED) and vertical-cavity surface-emitting lasers (VCSEL) are two types of suitable light sources. While VCSELs offer an attractive option for monolithic integration, their collimated light output may not be desirable, compared to the diffused light from LEDs [59]. Due to a wide selection of wavelengths available, LEDs are often the best choice for integrating lighting on-chip. Previously we experimented with illumination using a light fiber guided into tissue, but illumination was non-uniform and not repeatable enough to be useful for quantitative imaging. In order to improve the signal-to-noise ratio, filtering of the excitation light is necessary. Dandin *et al.* [60] reviewed the technologies available for incorporating optical filters onto integrated sensors. Interference filter is the best choice due to high wavelength selectivity of transmitted light. However, the process variations can cause large errors in cutoff wavelengths. An organic absorption-based color filter can be easily applied onto the sensor surface. Finally, a multiple junction photodiode structure can be used to discern different wavelengths of incident light [61-62]. The transmittance of specific wavelengths and hence effectiveness of these aforementioned methods decreases, with interference filter being the best option and multiple junction photodiodes being least efficient method. However, there are other tradeoffs, including ease of fabrication that must be taken into account. We used an organic filter which can be easily coated onto the sensor surface. Wavelength transmittance of around 44 dB can be achieved this way [40].

Sealing or encapsulating the implantable device to protect it from the corrosive environment of tissue is another important issue. Al is normally used as the interconnect material in standard CMOS processes. Because Al is not biocompatible, it is necessary to form a barrier layer between Al and the surrounding tissue. One method interesting method implemented by Heer *et al.* [63] is to use a shifted metal connection and forming a biocompatible electrode on the surface. Another way is to seal-off the exposed Al with a transparent and water impermeable epoxy while exposing the bump electrodes during the epoxy curing process [64]. This is accomplished by using a flat silicone layer placed on top of the device. Both methods are equally feasible, although the latter method requires less processing steps.

**Figure 7.**
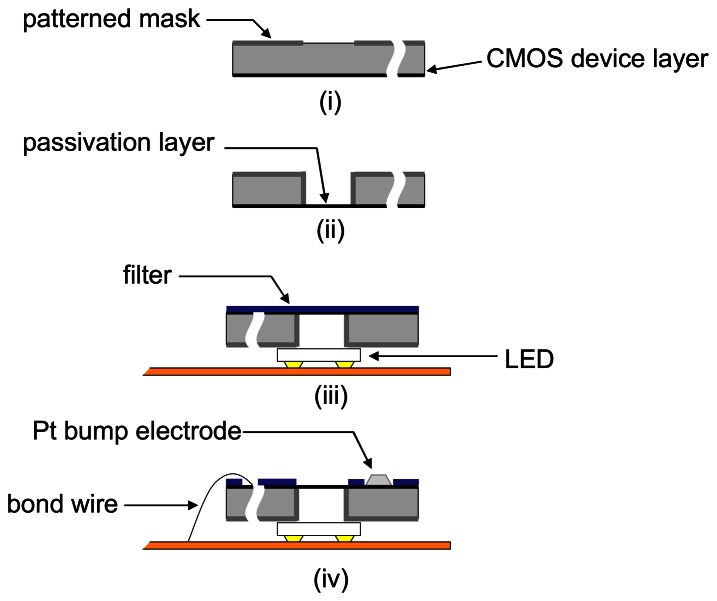
Post-processing of the sensor chip and packaging process flow of implantable device. (i) Pattern Al as mask for DRIE on backside of sensor chip, (ii) deep reactive ion etch backlit vias and sensor curved outline, (iii) attach sensor chip on top of flip-chip bonded LED, and spin coat optical filter, (vi) laser-assisted ablation of resist at bond sites followed by wire bonding of input output pads and forming Pt electrode onto Al electrodes. Finally device is sealed with transparent epoxy and precision laser cut out final shape (adapted from [11]).

A typical process for postprocessing and packaging a microimager for implantation is shown in Figure 7. By etching from the underside of the sensor chip, we can define backlit vias for illumination through the sensor. This allows for more compact arrangement of the LEDs as the illumination source. Also, a uniformly arranged backlit vias can give a fairly uniform illumination at the sensor surface. This is important for on-chip imaging due to the limitation of dynamic range of the image sensor.

## 4. System Integration

The microimager needs to be designed for final integration into an imaging and measurement system. In the design stage, the number of interconnections from the device to external interface is an important factor to be considered. The higher the number of connections, the more complicated will be the interface. This will lead to difficulties when performing experiments involving moving animals. One way to reduce the number of interconnections is to reduce the number of input-output pads on the microimager. For data transmission, a single serial data interface is adopted. Also, different bias voltages necessary for the amplifiers and buffers can be pulled down from the voltage supply line by using suitable on-chip resistors. Finally, by optimizing the layout of the row and column scanners, the clock and reset signals can be supported by a single clock signal input. Hence, including the power supply and ground line, only four connections to the chip is needed.

The importance of software development as part of the entire system cannot be over emphasized. Real-time graphing and analysis of data are important for decision making during *in vivo* imaging experiments. Also, the software needs to accommodate requests for data acquisition to be paused, stopped, and restarted during the entire experiment. Hence, a means to store and restore initial conditions is important. Apart from that, a user friendly interface will promote the use of the system to a wider audience. We have experimented with different ways to process data, both on-line and of line, graphical and textual, with different compression strategies. This led to a robust data and imaging storage system. Because behavioral imaging experiments can run for the duration of more than a week, the software must be flexible and adapt to the total amount of experiment time. Apart from monitoring the data from the imager over a network, vital signs need also be monitored together. This is especially important for freely moving animal experiments. Also, triggers and alarms are necessary to be reduce direct user monitoring time.

## 5. Animal Testing

In order to verify the functions of the implantable microimagers, experiments using animals need to be performed. These experiments involve small animals such as frogs, rabbits and mice which are anesthetized initially during device insertion but are awake during the experiments. Surgical procedures are performed under strict animal handling guidelines, by trained professionals.

### 5.1 Retinal Stimulation

**Figure 8.**
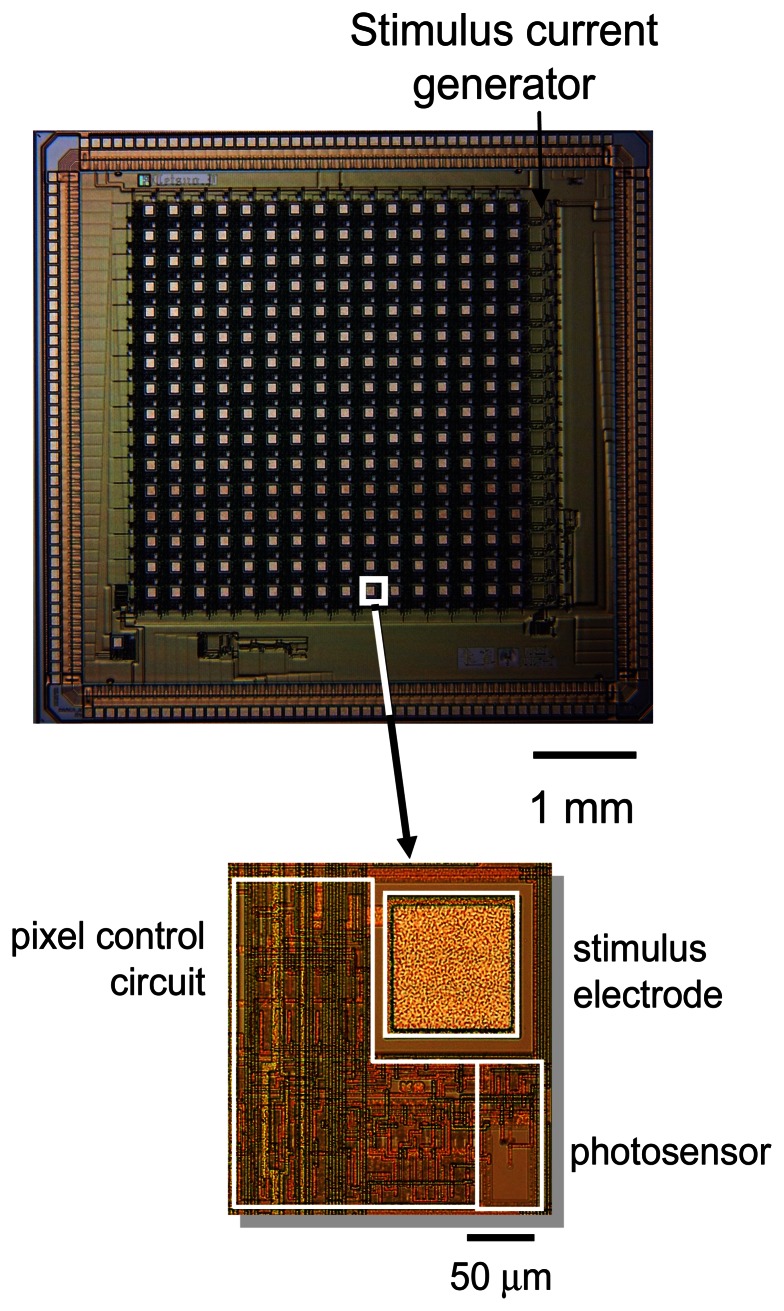
Retinal prosthesis chip showing close-up of single pixel (adapted from [65]).

We designed a prototype microimager device for retinal stimulation. It consists of a 16×16 pixel array, with each pixel containing a photosensor and stilumus electrode site. Figure 8 shows the retinal prothesis implant prototype chip and its corresponding specification is listed in Table 2 [65-66]. The photosensor used in this prototype chip is based on pulse frequency modulation (PFM) photosensing circuit. The PFM photosensor converts input light into a pulse train, and the frequency of the output pulse varies in proportion to the light intensity. The chip is equipped with a 3-bit programmable current generator which is capable of generating stimulus current beyond the threshold necessary to evoke a retinal response.

**Figure 9.**
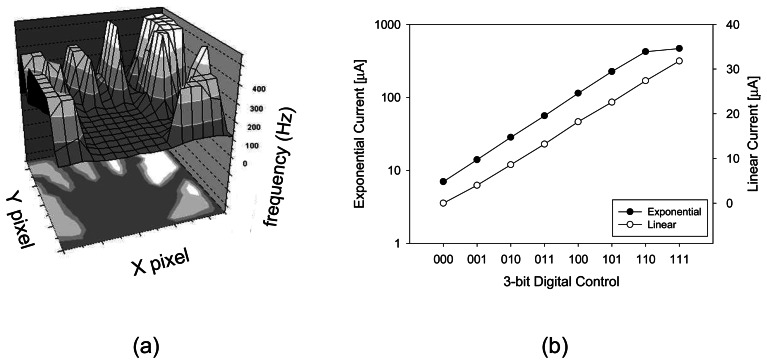
(a) Visualization of pulse output from 16×16 pixel array retinal prosthesis prototype chip based on pulse width modulation detection. (b) Stimulus current generated from a single electrode measured in saline (adapted from [65]).

We have tested the retinal prosthesis module *in vitro* using the detached retina of a frog and found that the firing rate of retinal ganglion cells increases in respond to incident light intensity. Figure 9 (a) shows the visualization of pulse output from the pulse frequency modulation-based retinal prosthesis chip. A backlit object illuminates the image sensor and region with higher light intensity shows a corresponding high frequency output pulse. The stimulus current can be controlled to vary linearly or exponentially as shown in Figure 9 (b). This increases the output range while ensuring that small current increments can be precisely controlled.

**Figure 10.**
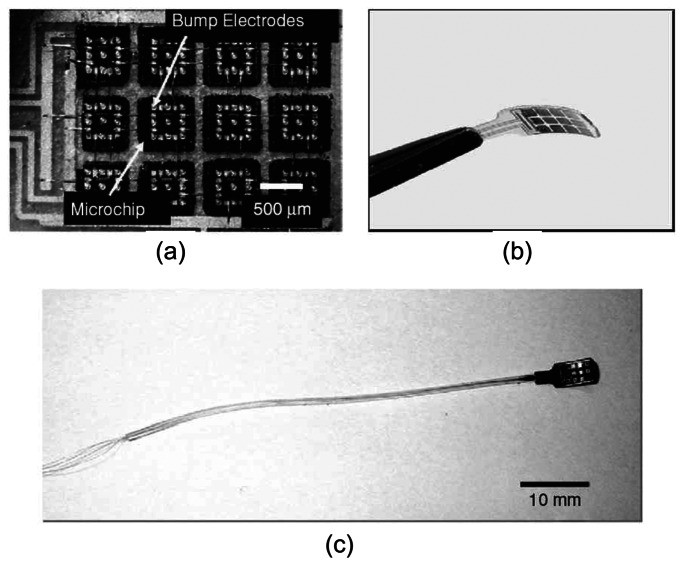
Photographs of a fabricated microchip-based stimulator for retinal prosthesis. (a) close-up of the microchips, (b) bending of the stimulator, and (c) the stimulator with platinum wires covered with silicone tubing [64] (© 2006 IEEE).

Although the functions of the prototype retinal prosthesis chip were verified, application in the implanted state presents further challenges. The device needs to be flexible enough to fit the anatomical structure of the eyeball to avoid damaging tissues. One way is to lap the device until it is thinner than 50 μm. However, this results in a very fragile device. In another approach, a microchip stimulator device was developed with distributed electrode unit architecture [38, 67]. This results in a flexible and extendible device (Figure 10). Current progress is being made in using this device for *in vivo* testing in the eye of the rabbit.

### 5.2 Functional Brain Imaging

**Figure 11.**
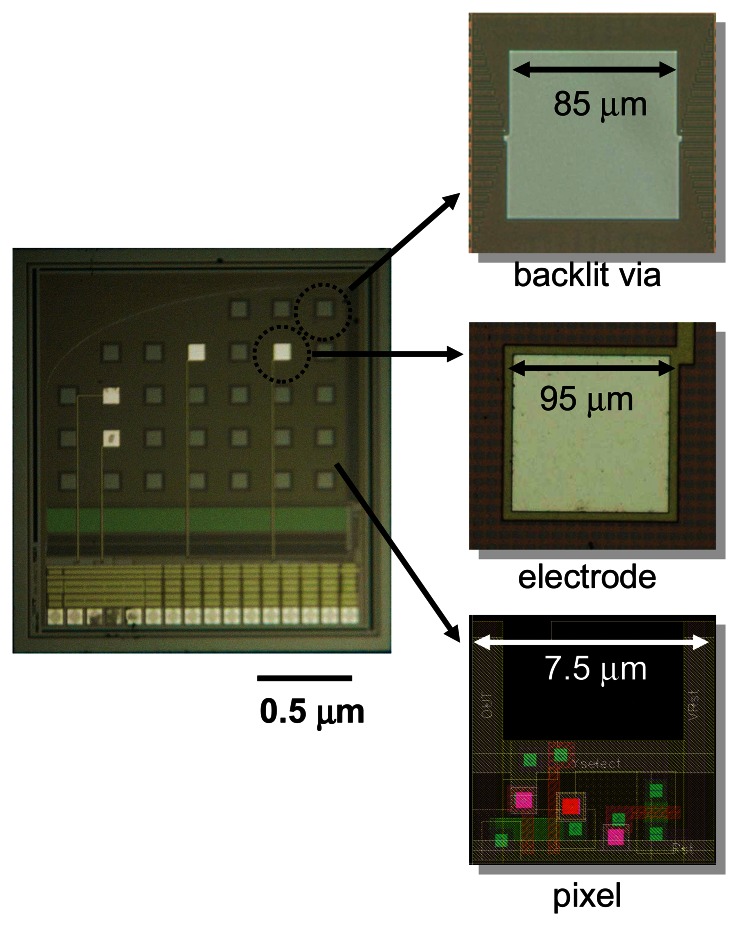
*In vivo* microimager chip showing close-up of backlit via, electrode and pixel (adapted from [11]).

**Figure 12.**
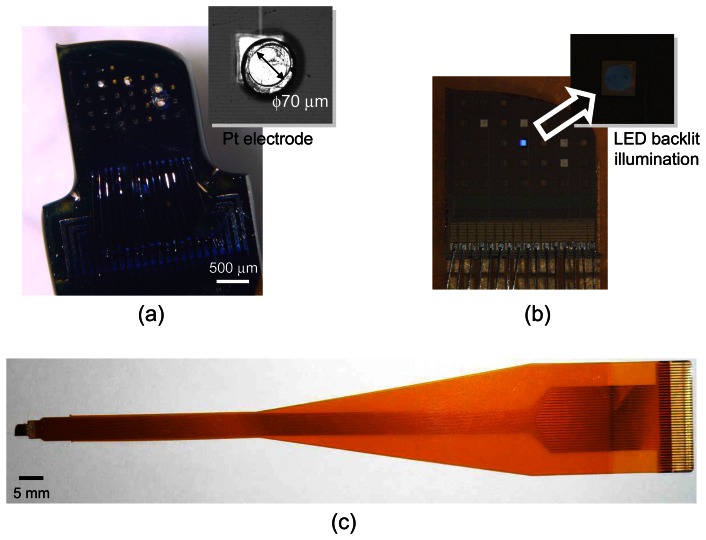
Microphotograph of fully packaged in vivo imager module. (a) close-up showing the image sensor and electrodes (inset), (b) LED illumination from integrated LED located under the sensor, and (c) full view of the device which is packaged onto a flexible polyimide substrate with printed interconnects (adapted from [11]).

Figure 11 shows a microimager device designed with backlit vias and electrodes for simultaneous fluorescence imaging and electrical interfacing with the neurons of a mouse brain. The device specification is listed in Table 3. Embedded within the imaging array are four electrodes placed at strategic positions for electrical stimulus and recording in the hippocampus of the mouse. These electrodes are used to provide electrical stimulus to the Schaffer-collateral pathway of the mouse hippocampus and record neuronal responses in the form of electric field potential. The device front surface is hyperbolically curve to reduce contact area between the device and tissue during insertion in order to minimize damage to the tissue. The backlit vias are uniformly places to ensure uniform light distribution on the sensor surface. Figure. 12 shows the fully packaged microimager module. An injection needle was attached to the module for injection of chemicals close to the sensor surface.

**Figure 13.**
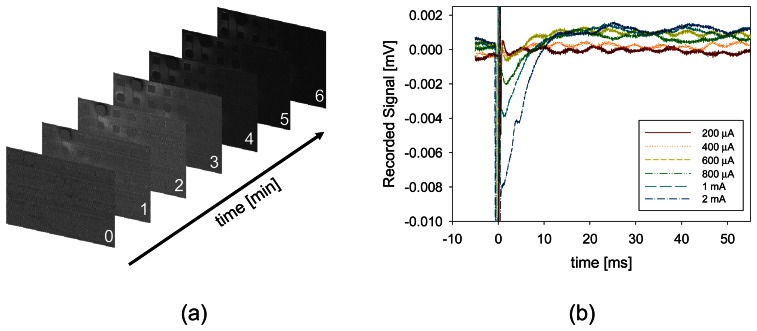
(a) Fluorescence images captured after high frequency theta-burst stimulation in the hippocampus showing increase in protease activity. (b) Electric field potential recordings at various stimulation current strength by using the on-chip Pt electrodes.

An experiment was performed to verify fluorescence imaging *in vivo* using the packaged module. In the experiment Boc-Val-Pro-Arg-4-methylcoumarin-7-amide (VPR-MCA), a fluorescence substrate was first injected into the hippocampus. Using a syringe pump, 2 mM of VPR-MCA in artificial cerebrospinal fluid (ACSF) was injected at 0.08 ml/min for 20 min. A high frequency pulse train (theta-burst) stimulation was then applied through the electrodes. This caused a chain of chemical response which leads to extracellular expression of neuropsin, a serine protease linked to the learning and memory processes in the brain. The serine protease reacts with the fluorescence substrate to release the bound fluorophore molecule. When illuminated by excitation light from the LEDs the molecule emit fluoresce light which were captured by the device as shown in Figure. 13 (a).

Another experiment was performed to verify the stimulus and recording capability of the on-chip electrodes. The device was inserted until the on-chip Pt electrodes were positioned at the Schaffer-collateral region. Two Pt electrodes were used for bipolar stimulation. A single monophasic 100 μs pulse was injected through one of the Pt electrodes and the other acted as the return path to close the electrical circuit. Synaptic response in the form of field potential recordings was measured by using the remaining electrodes. Figure 13 (b) shows the recorded signal at various stimulus current intensities. These recordings represent field excitatory post-synaptic potential responses which were elicited from the dendrites in the CA1 region when the Schaffer-collateral was stimulated. These responses are due to membrane depolarization of the post-synaptic terminal and are manifested by negative peaks in the recorded signal. Together, these results confirm the capability of the device, both for fluorescence imaging and neural interfacing.

## 6. Conclusion

The development of implantable microimagers is relatively new compared to other more advanced devices such as cochlear implants. In this paper, we have looked at the various aspects of design, packaging, and integration of microimagers for implantation inside the head. Looking at the level of development so far, we are convinced about the potential of these devices for other applications such as treatment of brain disorders and cancer diagnosis. We are continuing our efforts in developing an imaging platform for use in freely-moving animals. Data telemetry and power delivery are some of the expected challenges in designing future devices.

## Figures and Tables

**Table 1.** Specifications of microimager chip (adapted from [40]).

Technology		0.35 μm std. CMOS 4M2P
Operating voltage		3.3 V
Chip size		2 mm×2.2 mm
	
Image pixel	type	modified 3-transistor APS
	number	176×144
	size	7.5×7.5 μm^2^
	
Photodiode	type	Nwell-Psub
	size	16.2 μm^2^
	
Image sensor output		Serial analog voltage

**Table 2.** Specifications of retinal prosthesis chip (adapted from [65]).

Technology		0.6 μm CMOS (2-poly 3-metal)
Voltage source		3 V (logic), 5 V (stimulus)
Chip size		6 mm×5.6 mm
	
Pixel	size	240 μm×240 μm
	count	16×16
	
Photodiode		n-well/p-substrate
Electrode size		100 mm×100 mm
Amplitude resolution		3-bit exponential, 3-bit linear (biphasic)
Base clock		2 MHz
Serial communication speed		500 kHz
Frame rate		4 to 8,000 Hz

**Table 3.** Specifications of *in vivo* microimager (adapted from [11]).

Technology		0.35 μm std. CMOS 4M2P
Operating voltage		3.3 V
Chip size		2 mm×2.2 mm
	
Image pixel	type	3-transistor APS
	number	224×164
	size	7.5×7.5 μm^2^
	
Photodiode	type	Nwell-Psub
	size	19.75 μm^2^
	
Electrode	size	90×90 μm^2^
	number	4
	
Image sensor output		Serial analog voltage
